# Overexpression of Platelet-Derived Growth Factor Receptor Α *D842V* Mutants Prevents Liver Regeneration and Chemically Induced Hepatocarcinogenesis via Inhibition of MET and EGFR

**DOI:** 10.7150/jca.44492

**Published:** 2020-05-18

**Authors:** Zhao-Qing Du, Jian Dong, Mu-Xing Li, Jian-Fei Zhang, Jian-Bin Bi, Yi-Fan Ren, Li-Na Zhang, Rong-Qian Wu, Satdarshan P.S. Monga, Yi Lv, Xu-Feng Zhang, Hai-Chen Wang

**Affiliations:** 1 Department of Hepatobiliary Surgery and Institute of Advanced Surgical Technology and Engineering, The First Affiliated Hospital of Xi'an Jiaotong University. Xi'an, Shaanxi Province, 710061, China;; 2 National-Local Joint Engineering Research Center for Precision Surgery & Regenerative Medicine, The First Affiliated Hospital of Xi'an Jiaotong University. Xi'an, Shaanxi Province, 710061, China;; 3 Department of General Surgery, Peking University Third Hospital, Beijing, 100083, China;; 4 Department of Surgical Oncology, Shaanxi Provincial People's Hospital, Xi'an, 710068, China;; 5 Department of Pharmacy, the Second Affiliated Hospital of Xi'an Jiaotong University, Xi'an, China;; 6 Department of Pathology and Medicine and Pittsburgh Liver Research Center, University of Pittsburgh, School of Medicine and University of Pittsburgh Medical Center, Pittsburgh, PA, USA;; 7 Department of Cardiovascular Surgery, The First Affiliated Hospital of Xi'an Jiaotong University. Xi'an, Shaanxi Province, 710061, China.

**Keywords:** platelet-derived growth factor receptor α, liver, transgenic, hepatocyte growth factor receptor, epidermal growth factor receptor

## Abstract

Platelet-derived growth receptor α (PDGFRα) is a key factor in many pathophysiological processes. The expression level of PDGFRα is significantly elevated in the early stage of liver development and maintained at a lower level in adult normal livers. In this study, we constructed a liver-specific PDGFRαD842 mutant transgenic (TG) mice model to explore the effect of continuous activation of PDGFRα on liver regeneration and hepatocarcinogenesis. 14-day-old TG and wild-type (WT) mice were intraperitoneally injected with diethylnitrosamine (DEN) at a dose of 25 μg/g body weight. Two-month-old male TG and WT mice were subjected to partial hepatectomy (PH). The liver tissues were collected for further analysis at different time points. Overexpression of PDGFRα*^D842V^* and its target genes, Akt, c-myc and cyclin D1 in hepatocytes with no overt phenotype versus WT mice were compared. Unexpectedly, a dramatic decrease in hepatocyte proliferation was noted after PH in TG versus WT mice, possibly due to the downregulation of hepatocyte growth factor receptor (MET) and epidermal growth factor receptor (EGFR). No TG mice developed HCC spontaneously after 14 months follow-up. However, TG mice were more resistant to DEN-induced hapatocarcinogenesis at 6, 10, and 12 months of age, showing delayed hepatocyte proliferation and apoptosis, lower tumor incidence, smaller size and fewer number, compared with age-matched WTs, partially through downregulation of MET and EGFR. In conclusion, continuous activation of PDGFRα signaling by expression of PDGFRα*^D842V^* does not promote, but inhibit hepatic regeneration and hepatocarcinogenesis, possibly through compensatory downregulation of MET and EGFR.

## Introduction

Platelet-derived growth receptor (PDGFR) belongs to the tyrosine kinase receptor ІІІ family and contains two types of receptors, PDGFRα and PDGFRβ. PDGFRs bind to their ligand PDGFs (PDGF-A, B, C, D) in the form of homodimer or heterodimer, then the tyrosine staging of the receptor is induced to further activate the downstream signal pathways and produce a series of biological effects. PDGFRα binds primarily to PDGF-A or PDGF-C in the form of homo- or heterodimers.^1^ Activation of PDGFRα and downstream signaling, phosphatidylinositol 3-kinase (PI3K)/Akt, Ras/MARK and Phospholipase C-gamma (PLC-γ), have been implicated in the various pathophysiologic process. 1-4

Interestingly, PDGFRα might play an indispensable role at the early stage of embryonic growth and development, while after birth it was reduced to a non-major factor.^2^ However, activation of PDGFRα signaling has been verified in the development and metastasis of many types of cancers, including hepatocellular carcinoma (HCC). 1, 5-7 Specifically, increased expression of PDGFRα has been found in about 65%-80% cases of patient HCC versus adjacent non-tumorous tissues. 2, 5, 6, 8 Overexpression of PDGFRα in vascular endothelial cells was found to be positively associated with metastasis and recurrence of HCC.^9^ Thus, PDGFRα is considered as an “oncogene” and a new target for the treatment of HCC.^1^ However, whether modulation of PDGFRα expression in hepatocytes would have any impacts on liver pathophysiology remains largely unknown.

PDGFRA gene activation was frequently found in 82% to 93% cases of gastrointestinal stromal tumors (GIST).^10^ And a small case of PDGFRA gene (~5%) expressed the point mutation, so PDGFRα was in a sustained activation state.^10^ Specifically, PDGFRA mutations are found mostly in exons 18 (tyrosine kinase 2 (TK2) domain). And mutation for D842V in exon 18 is resistant to imatinib.^11^ The D842V mutation results in an amino acid substitution at position 842 in PDGFRA, from an aspartic acid (D) to a valine (V). This mutation occurs within the TK2 domain.^11^ Kurth et al. constructed organism nonspecific transgenic mice using the sustained activation of PDGFRα mutant (D842V) in GIST.^12^ Olson et al. established two non-specific transgenic mice by using two PDGFRα mutants (D842V and V561D), which were common in human GIST.^13^ However, this method tended to produce defects or multiple organs dysfunctions in the development of transgenic models.^12^, ^13^ Hence, specific-organ gene transfection provides the best option for exploring the significance of a gene in the physiology and pathology, because it can be deleted or transfected into a particular gene in a particular organ without causing the molecular signal mechanism changes of the other organs and tissues, which will lead to a higher survival rate of the models.

To deeply explore the role of PDGFRα in the development of liver regeneration and HCC, we constructed a liver-specific PDGFRα*^D842^*mutant transgenic (TG) mice model. When subjected to partial hepatectomy (PH) or N-nitrosodiethylamine (DEN) induction, PDGFRα*^D842^*TG mice displayed decreased liver regeneration capacity and lower tumor development compared with sex - and age-matched wildtype (WT) mice. Further studies identified that the downregulation of hepatocyte growth factor receptor (MET) and epidermal growth factor receptor (EGFR) might be a pivotal mechanism counteracting the overexpression and activation of PDGFRα*^D842V^*, which led to delayed and compromised liver regeneration after PH and decreased HCC development in TG mice.

## Materials and Methods

### Generation of PDGFRα*^D842V^* conditional transgenic mice

**Figure 1:**
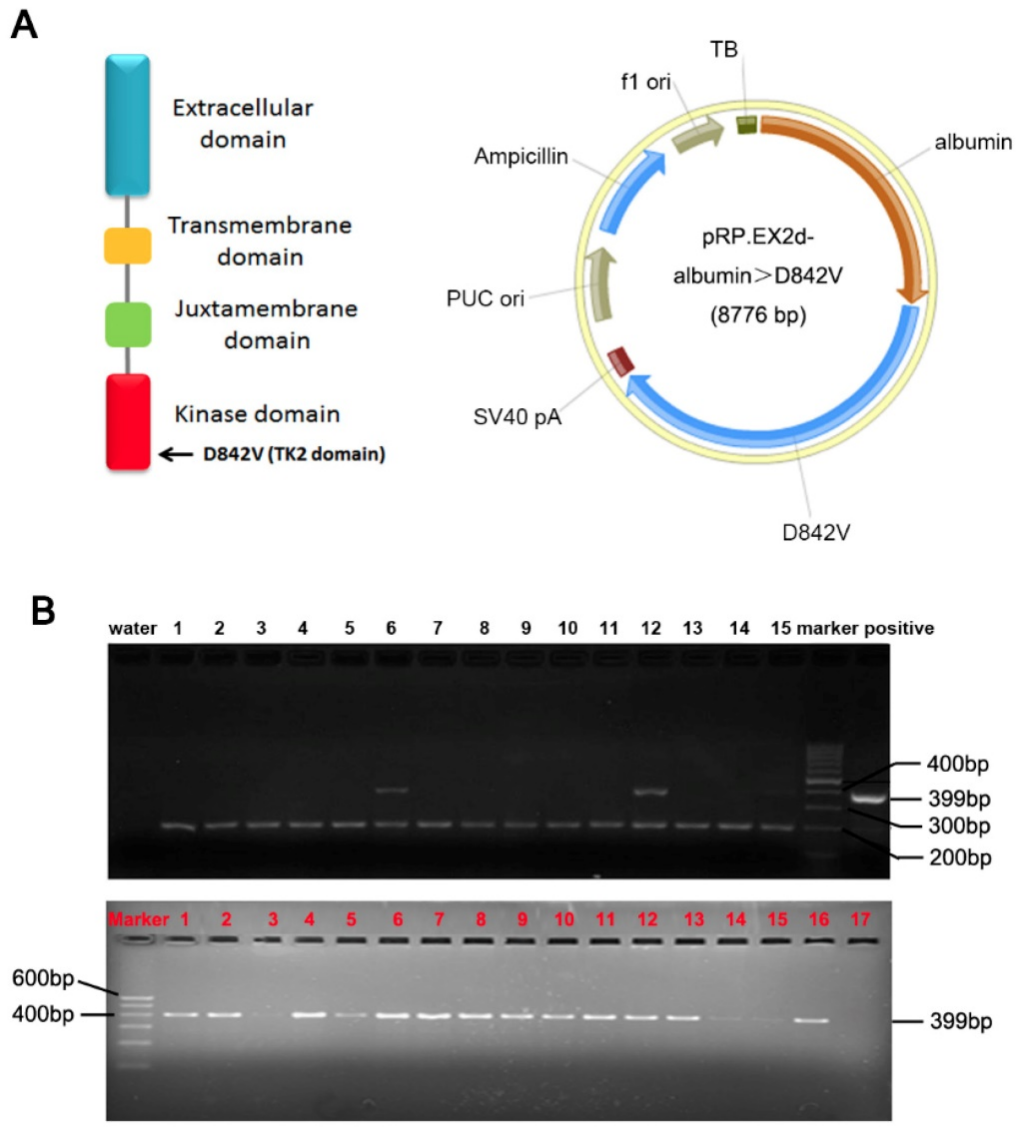
** Construction of PDGFRα*^D842V^* transgenic mice (TG).** (A) A transgenic Prp.EX2d-Albumin-D842V plasmid which was induced by an albumin promoter/enhancer-driven expression vector with 8776bp mouse PDGFRα gene, was used to construct TG mice through microinjection. (B) Positive F (0) founders (Upper panel) and TG littermates (Lower panel) were judged by PCR analysis. As identified, No. 6 and No. 12 are positive F (0) founders. The top bands were the target genes (399bp), while the bottom bands (200bp) were from control primers targeting an endogenous site in the mouse genome.

A PDGFRα D842V mutation was introduced by site-directed mutagenesis of a human wild-type PDGFRα cDNA. The albumin promoter/enhancer regulatory expression vector has been constructed as we previously stated.^5^, ^7^, ^14^ The PDGFRα*^D842^*cDNA was inserted into the *Bam*HI sites of the albumin promoter/enhancer-driven expression vector to generate the transgenic pRP.ExSi-Albumin-PDGFRα*^D842V^* plasmid (Figure 1A). The albumin PDGFRα*^D842V^* expression vector was linearized and microinjected into C57BL/6×SJL hybrid mice fertilized egg cells. Then the genomic DNA of mice tail was extracted and the genotype was identified through polymerase chain reaction (PCR) analysis. The primer for PDGFRα was: 5'-GAG CAC AAG AAG TTA TGT GAT TTT G-3'; 5'-CCA TGA TCT CAT AGA CTT CAC TGG T-3'. The size of the PCR product strand in the transgenic mice was 399bp. TG mice were crossed with C57BL/6 mice to obtain F1 heterozygous mice, and then bred further to obtain heterozygous TG mice. Only male TG littermates with stable overexpression of PDGFRα*^D842V^* were selected for further studies. All experiments on mice were done under strict management of the Institutional Animal Use and Care Committee at the Xi'an Jiaotong University.

### Animal treatment and specimen collection

Three-month old male PDGFRα*^D842V^* TG and WT mice were subjected to partial hepatectomy (PH), and sacrificed at 1 day (d), 2d, 3d, respectively (n≥4 at each time point). Livers were harvested for paraffin embedding and protein isolation.

Another cohort of male PDGFRα*^D842V^* TG and WT mice were selected, and a single intraperitoneal injection of diethylnitrosamine (DEN, Sigma, St. Louis, MO) (25 μg/g body weight) was performed at 14 days after birth. Both TG and WT mice were sacrificed at 6 months (m), 10m and 12m (n). The survival rate, tumor incidence, maximal nodule sizes and numbers of the two groups were documented in detail and compared between TG and WTs. The tumor tissues of the mice were collected and frozen in liquid nitrogen for the next protein and RNA analysis, and also fixed in 4% paraformaldehyde for 24 hours and subsequently embedded in paraffin.

### Real-Time RCR Analysis

Total RNA was extracted from mice liver tissues using the TRIzol Reagent system (Invitrogen, US). The extracted RNA was assayed for purity and concentration using an automatic microplate reader (Thermo Scientific) and then reversely transcribed using PrimeScript RT Master Mix (Takara) to obtain cDNA, and then real-time quantitative PCR was performed using SYBR-Green PCR Master Mix (Applied Biosystems; Grand Island, NY). The Applied Biosystems StepOnePlus Real-Time PCR System was analyzed with StepOne software version 2.1. The design and synthesis of all the primers involved in the experiment were performed by Takra Bio Inc. The detailed primer sequence is as follows: mouse PDGFRα forward: 5'-GCCGGTCCCAACCTGTAATG-3'; mouse PDGFRα reverse: 5'-AGGCTCCCAGCAAGTTCACAA-3'; mouse PDGF-A forward: 5'-GCGACTCTTGGAGATAGACTCCGTA-3'; mouse PDGF-A reverse: 5'-CGTAAATGACCGTCCTGGTCTTG-3'; mouse PDGF-C forward: 5'-CCCGGATTCTGCATCCACTAC-3'; mouse PDGF-C reverse: 5'-GTTGAGCAGGTCCAATGACAAAG-3'.

### Western Blot (WB) Analysis

The specimens were washed by cold PBS solution, the tissue cells were decomposed by cell lysate liquid, and protease inhibitor (Roche) and phosphatase inhibitor were added. The protein concentration was determined by the quinoline carboxylic acid method (BCA) (Pierce, Rockford, IL). The proteins were separated by 10% SDS-polyacrylamide gel electrophoresis and transferred to a PVDF membrane (Millipore, Billerica, MA). Sealed was done by TBS solution and 5% skimmed milk powder containing 0.1% Tween 20. Appropriate dilution of β-actin (Proteintech; HRP-60008, 1:5000), PDGFRα (R&D; AF1062, 1: 200), AKT1/2/3 (Santa Cruz; sc-8312, 1:1000), P-AKT (Thr308) (Cell signaling; 13038, 1:1000), cyclin D1 (Santa Cruz; sc-20044, 1:1000), C-myc (Santa Cruz; sc-40, 1:1000), MET (Santa Cruz; sc-8057, 1:1000), EGFR (Santa Cruz; sc-373746, 1:1000) of primary antibody in blocking buffer, and then the membrane was placed in a refrigerator overnight at 4°C. Removed the membrane and washed 3 times (10min per time). And added with the secondary anti-buffer (1: 5000, Pioneering Biotechnology, China), sealed at room temperature for 1h. At the end of the wash, the protein blots were detected by light emission (Millipore, Billerica, MA). Western blot grayscale values were quantified using Adobe Photoshop CS6 software.

### Histology and Immunohistochemistry

Masson Trichrome Staining, Ki67 and TUNEL were performed as described previously.^7^, ^14^ Ki67 and TUNEL positive hepatocytes were identified and documented using a microscope in ten randomly fields per section at a magnification of 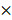
400. Masson Trichrome and PDGFRα immunohistochemical staining were performed using Image-Pro Plus 6.0 software for semi-quantitative analysis.

Continuous sections were embedded in paraffin with a thickness of 5μm, degreased by xylene, and rehydrated by 3% hydrogen peroxide. The main antibodies used in the experiment were PDGFRα (R&D; AF1062, 1: 100), CD31 (R&D; AF3628, 1: 100), Ki67 (Proteintech, 27309-1-AP, 1:50), AKT1/2/3 (Santa Cruz; sc-8312, 1:50), cyclin D1 (Santa Cruz; sc-20044, 1:50), C-myc (Santa Cruz; sc-40, 1:50), MET (Santa Cruz; sc-8057, 1:50), EGFR (Santa Cruz; sc-373746, 1:50). At room temperature, a biologically labeled secondary antibody was added, and transfected using streptavidin-biotin (SABC) complex. After that, it was stained with 3,3'-diaminobenzidine (DAB), then treated with hematoxylin, and observed the results under a microscope.

Apoptotic nuclei were detected by terminal deoxynucleotidyl transferase-mediated deoxyuridine triphosphate nick-end labeling (TUNEL) staining using ApopTag Peroxidase kit (Intergen Company).

All slides were viewed under a BX53F upright research microscope (Olympus) and digital images were obtained by Nikon Coolpix camera.

### Statistical Analysis

All data were analyzed using IBM SPSS statistical software (IBM Corporation, Armonk, NY, USA). Data were expressed as mean ± standard deviation (S.D.) and were analyzed using Student* t* test or ANOVA. *p*<0.05 was considered statistically significant.

## Results

### Construction of PDGFRα*^D842V^* overexpression mouse model

To investigate the role of PDGFRα during hepatic regeneration and carcinogenesis, we took advantage of the PDGFRα*^D842V^* mutation in the development of GISTs. This point mutation of PDGFRα leads to auto-activation of PDGFRα signaling even in the absence of ligands. 13 of the 72 pups (8 males and 5 females) were identified as positive PDGFRα*^D842V^* transgenic mice by PCR analysis and they were treated as F0 founders (Figure 1B, upper panel). F0 founder TG mice with significantly robust expression of PDGFRα were crossed with wild type C57BL/6×SJL mice to obtain F1 heterozygous mice, and they were further reared to obtain heterozygous transgenic mice for subsequent experimental studies (Figure 1B, lower panel).

**Figure 2:**
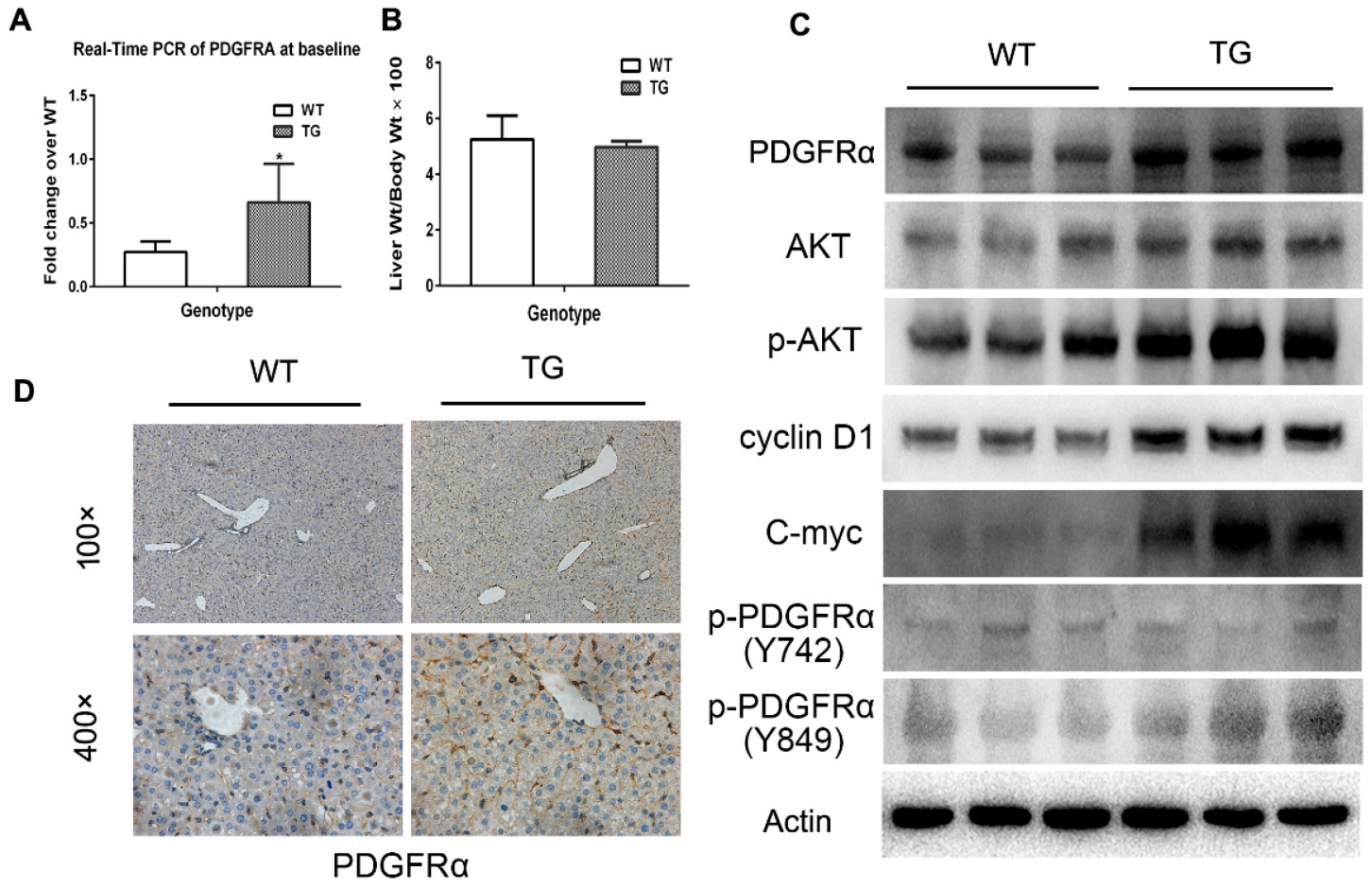
** Characterization of PDGFRα*^D842V^* transgenic mice (TG).** (A) Fold changes of PDGFRA expression in 2-month-old TG and WT mice. (B) Two-month-old TG mice and WT mice were randomly selected to obtain the LW/BW ratios. (C) PDGFRα expression in hepatocytes was detected by immunohistochemistry in TG and WT livers. (D) Western blot analysis investigated the upregulation of PDGFRα, and downstream target molecules in TG versus WT mice. *p<0.05.

Expression of PDGFRA in 2-month old TG mice was robustly increased compared with age-matched WT mice (Figure 2A), while the liver weight/body weight ratios (LW/BW) were comparable between TG and WT mice (Figure 2B). The expression of PDGFRα protein in TG livers was also dramatically increased versus WTs (Figure 2C and D). On immunostaining, increased expression of PDGFRα in TG livers was mainly located on the cell membrane and cytoplasm of the hepatocytes (Figure 2D). To further confirm the activation of PDGFRα signaling, we examined the major molecular proteins in the PDGFRα downstream pathway. It was found that phospho-PDGFRα at Y849 was elevated in TG livers versus WTs, which is required for the phosphoinositide 3-kinase (PI3K)/Akt signaling activation.^15^ In contrast, phospho-PDGFRα at Y742 remained unchanged between TG and WT livers, which is a contributor for Ras and Erk activation (Figure 2C).^15^ Similarly, expression of the downstream proteins, such as total Akt, phosphor-Akt, cyclin D1 and c-myc were all increased in TG versus WT livers (Figure 2C). These findings confirmed the generation of liver-specific PDGFRα transgenic mice and were subjected to further studies.

### Activation of the PDGFRα signaling pathway hampered liver regeneration after partial hepatectomy

**Figure 3:**
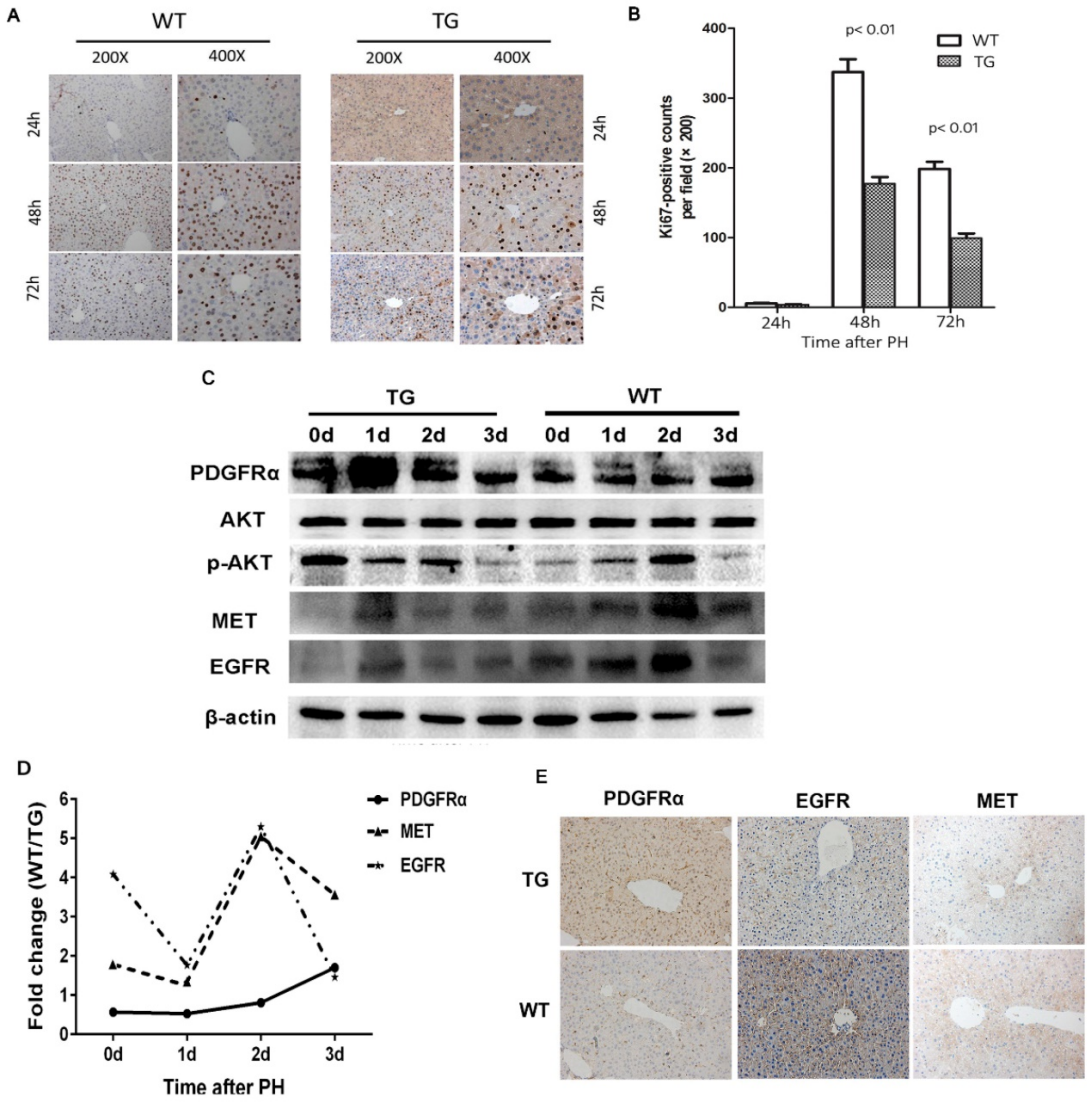
** Overexpression of PDGFRα*^D842V^* slowed liver regeneration by inhibiting EGFR and MET after partial hepatectomy (PH).** (A) Immunohistochemical analysis of Ki67 expression at 24 h, 48 h and 72 h after PH in TG and WT mice. (B) 5 fields were randomly selected from Ki67 staining per group to count the number of positive cells at each time point. (C) Western blot analysis investigated the expression of PDGFRα, MET, and EGFR in the TG and WT mice after PH at each time point. (D) Photoshop software was used to quantify the gray values of western blot bands of PDGFRα, MET and EGFR expression in WT versus TG after PH. (E) Immunostaining for PDGFRα, MET and EGFR in TG and WT livers after PH.

The molecular signaling involved in liver regeneration might be quite similar tothose in tumorigenesis.^16^ As such, we firstly studied the impacts of PDGFRα mutation and activation on liver regeneration after 2/3 PH. Liver specimens were obtained at 0, 1, 2, and 3 days after PH for age-matched TG and WT mice (n=4 for each group at each time point). IHC for Ki67 was performed to address the mechanism of enhanced mitosis in mice after PH. The peak hepatocytes proliferation after PH occurred at 48 hours among both TG and WT livers (Figure 3A and B). Intriguingly, the number of positive nuclei stained by Ki67 representing hepatocytes in S-phase was comparable in TG versus WT livers at 24 hours after PH, but significantly lower than in TG versus WT livers at 48 and 72 hours after PH (Figure 3A and B), which indicated an impaired regeneration capability of the liver after PDGFRα*^D842V^* overexpression.

To investigate the impacts of PDGFRα*^D842V^* overexpression on liver regeneration, we assayed for PDGFRα and its target genes, as well as other related tyrosine kinase receptors. A robust increase of PDGFRα was evident at 24 hours after PH in TG, but 72 hours after PH in WTs (Figure 3C). The level of PDGFRα was lower in WT versus TG before 48 hours after PH, but intriguingly even higher in WT than TG at 72 hours after PH (Figure 3D). Although baseline phosphor-Akt (Thr308) was higher in TG than WT livers (0d), the level of phosphor-Akt (Thr308) decreased in TG after PH, but increased 24 and 48 hours after PH in WT (Figure 3C). These findings were inconsistent with when compared with normal TG and WT (Figure 2D). We further evaluated the change of MET and EGFR. It was evident that the level of MET and EGFR in TG was lower than that in WT at baseline and each time point after PH (Figure 3C, D, and E). After PH, the level of MET and EGFR increased at a peak level at 24 hours in TG but 48 hours in WT (Figure 3C). These interesting results implied that overexpression of PDGFRα*^D842V^* delayed hepatic regeneration after PH, possibly via inhibition of other tyrosine kinase receptors, such as MET and EGFR.

### Increased expression of PDGFRα in mice prevented hepatocarcinogenesis

**Figure 4:**
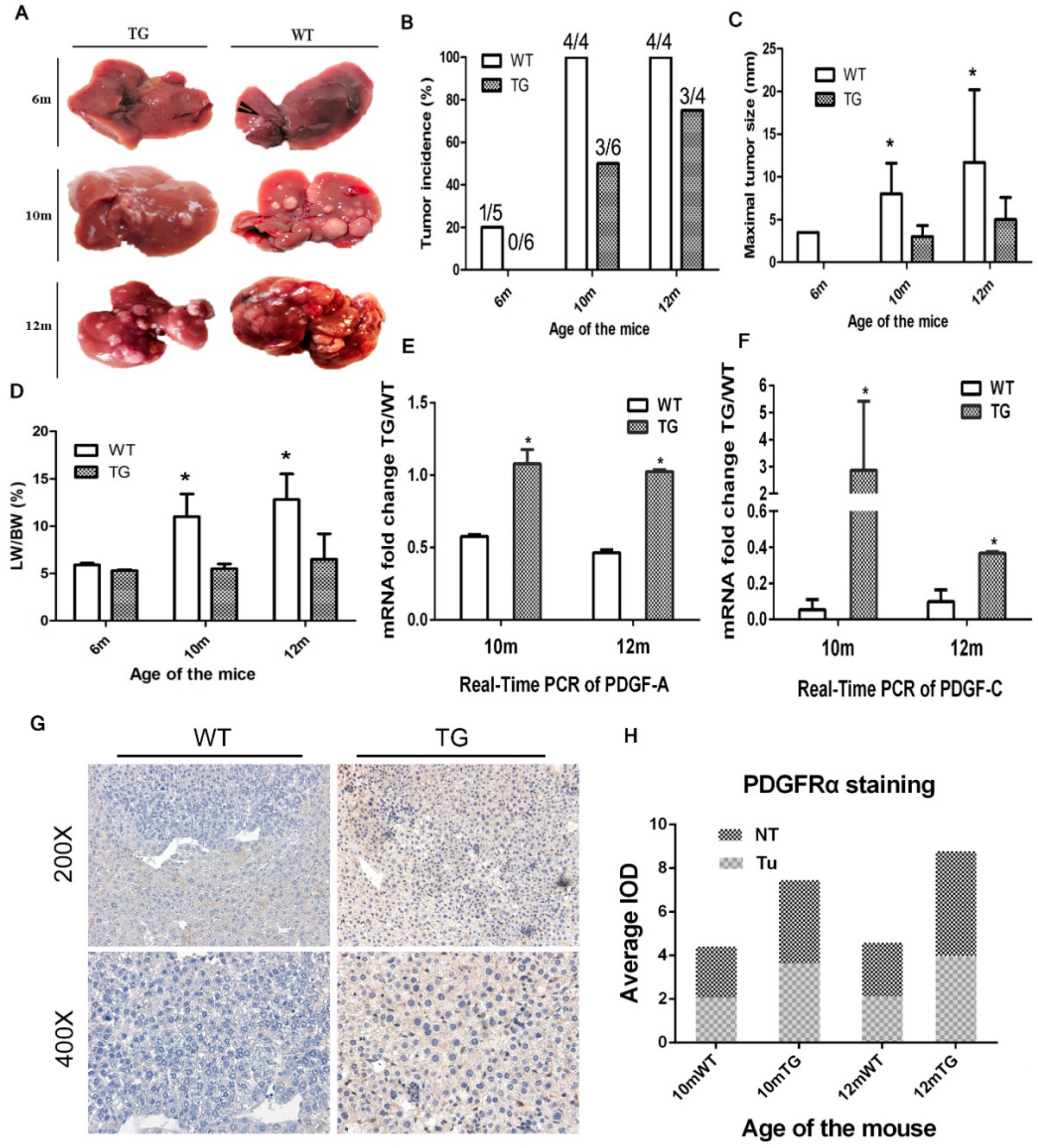
** Overexpression of PDGFRα*^D842V^* in TG mice inhibited hepatocarcinogenesis at 6, 10 and 12months after DEN exposure.** (A) Representative tumors of TG and WT livers at different months of age. (B) The incidence of hepatic tumors at different ages in WT and TG mice. (C) The maximum tumor size of WT and TG mice at different months of age. (D) LB/WB ratios at different ages in DEN-treated WT and TG mice. (E) & (F) Quantitative Real-Time PCR assayed the expression of two major PDGFRα ligands, PDGF-A (E) and PDGF-C (F), in TG and WT mice at 10 and 12 months after DEN administration. (G) & (H) Immunostaining for PDGFRα in TG and WT liver tumors and adjacent tissues. *p
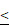
0.05.

TG mice were followed up for 14 months. No phenotypic abnormalities and spontaneous liver tumors were found. A total of 29 age-matched male mice were selected for a single intraperitoneal injection of DEN at a dose of 25μg/g body weight and they were randomly sacrificed at 6 months, 10 months and 12 months to obtain liver and tumor tissue. Interestingly, the incidence of liver tumors in the TG group was significantly lower than that in the WT group (Figure 4A and B). Specifically, the tumor incidence in the WT and TG groups were 20% and 0% at 6 months, 100% and 50% at 10 months, and 100% and 75% at 12 months, respectively (Figure 4B). Of note, the maximal tumor diameter in the WT group was significantly larger than that in the TG group at 10 and 12 months after DEN exposure (Figure 4C). A higher tumor load in WT than TG was also verified by a higher LW/BW in WT than TG mice at 10 and 12 months after DEN injection (Figure 4D).

Since there were no liver tumors in the TG mice at 6 months, only 10- and 12-month old mice were subjected for further mechanism studies. Two ligands of PDGFRα, PDGF-A and PDGF-C, were both elevated at 10 and 12 months in TG versus WT livers, which indicated a robust activation of PDGFRα signaling in TG livers (Figure 4E and F). IHC staining showed that PDGFRα was not only overexpressed in non-tumor tissues among TG versus WT livers, but more dramatically increased in TG tumors than WT tumors (Figure 4G and H). Taken together, PDGFRα*^D842V^* overexpression inhibited DEN-induced hepatocarcinogenesis. However, PDGFRα signaling activation appeared still as one of the major events in hepatocarcinogenesis in TG mice.

### PDGFRα TG livers after DEN injection prevented cell proliferation and apoptosis, and increased tissue fibrosis

**Figure 5:**
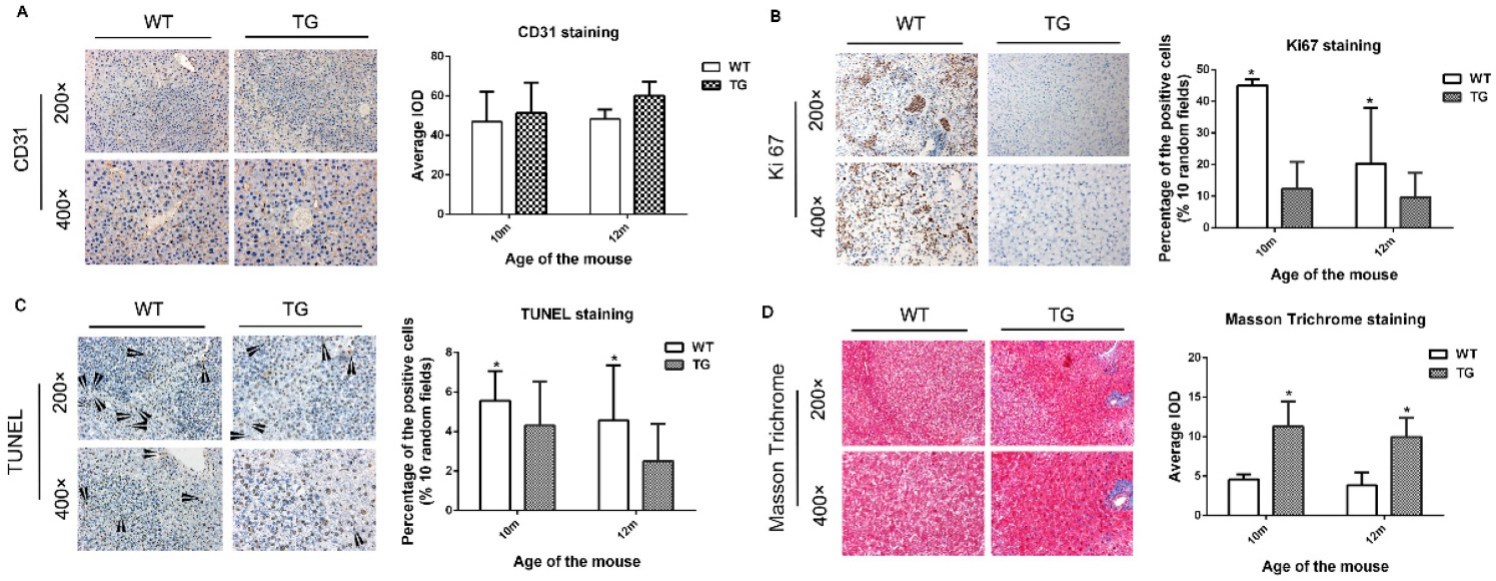
** Overexpression of PDGFRα*^D842V^* inhibited hepatocyte proliferation and apoptosis after DEN injection in TG mice, but promoting hepatic fibrosis.** (A) Immunostaining for CD31 showed comparable angiogenesis in TG and WT mice 10 and 12 months after DEN injection. (B) Immunostaining for Ki67 indicated that hepatocyte proliferation was less in DEN-treated TG versus WT mice at 10 months and 12 months of age. (C) TUNEL staining showed that hepatocyte apoptosis in TG livers was significantly decreased versus WT after DEN injection. (D) The Masson Trichrome staining confirmed that compared with WT, liver fibrosis in TG mice was significantly aggravated after DEN administration at 10 months and 12 months. *p<0.05.

Next, we further investigated the cellular effect of PDGFRα*^D842V^* overexpression on hepatic angiogenesis, hepatic fibrosis, cellular proliferation and apoptosis. On IHC staining for CD31, overexpression of PDGFRα*^D842V^* had no significant effect on tumor angiogenesis when compared between TG and WT livers (Figure 5A). In contrast, the number of Ki67-positive cells in DEN-treated TG livers was significantly lower than that in DEN-treated WT livers, which implied a retarded cellular proliferation in PDGFRα TG livers under DEN induction (Figure 5B). These findings were consistent with the results after PH (Figure 5B and 4A). TUNEL staining suggested that the numbers of apoptotic nuclei were significantly lower in TG than WT livers after DEN treatment (Figure 5C). In contrast, Masson Trichrome staining showed more severe tissue fibrosis in DEN-treated TG versus WT livers (Figure 5D). These findings suggested that decreased cellular proliferation and injury after PDGFRα*^D842V^* overexpression might partially account for the lower tumorigenesis in TG livers than WT livers.

### PDGFRα^D842V^ overexpression inhibited hepatocarcinogenesis after DEN injection through downregulation of EGFR and MET

**Figure 6:**
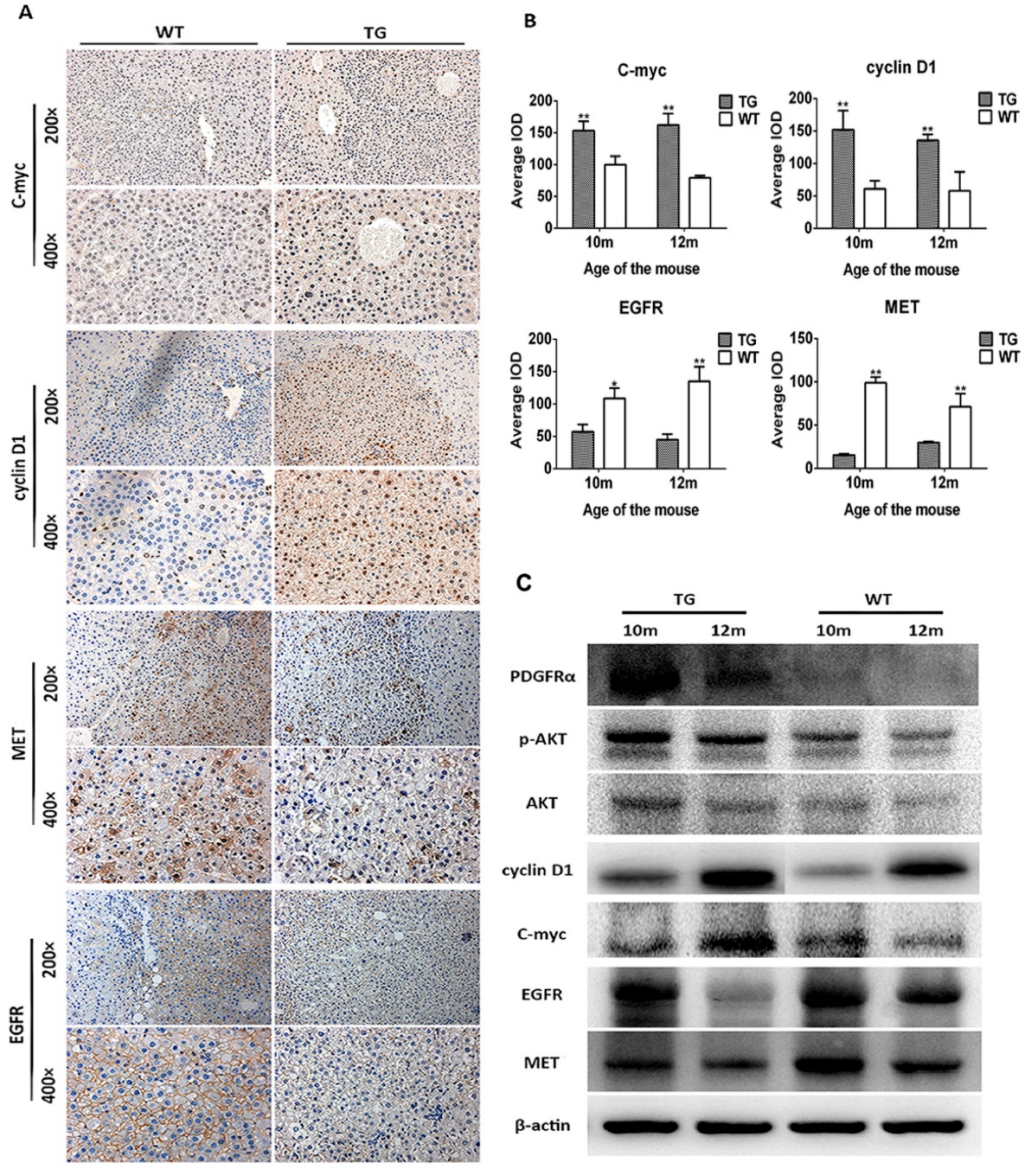
** Overexpression of PDGFRα*^D842V^* inhibited hepatocarcinogenesis by down-regulating MET and EGFR in TG livers after DEN administration.** (A) & (B) Immunostaining showed activation of PDGFRα downstream targets (c-myc and cyclinD1), but downregulation of MET and EGFR in DEN treated TG hepatic tumors, compared with WTs. (C) Protein assay for tumor tissues investigated activation of PDGFRα, physpho-akt, cyclinD1, c-myc, but decreased expression of MET and EGFR in TG versus WT tumors. *p<0.05, **p<0.01.

The mechanism of decreased tumor development in TG mice was further investigated. As a consequence of PDGFRα*^D842V^* overexpression, the downstream and target genes phosphor-Akt (Thr308), cyclin D1 and c-myc were upregulated in TG hepatomas compared with WT tumors on IHC staining (Figure 6A and B). In contrast, the level of MET and EGFR were both decreased in TG hepatomas versus WT hepatomas (Figure 6A and B), which was consistent with the findings in the liver regeneration model (Figure 3C and E). Protein analysis by WB also confirmed these findings that tumors in TG livers expressed a higher level of PDGFRα, phosphor-Akt (Thr308), cyclin D1 and c-myc, but lower level of MET and EGFR, although protein contamination by non-tumorous tissue might be not fully avoid when harvested because of the small size of each tumor (Figure 6C). Taken together, these findings implied that overexpression of PDGFRα*^D842V^* prevented DEN-induced hepatocarcinogenesis at least partially through the downregulation of other tyrosine kinase receptors, such as MET and EGFR. Among TG livers, PDGFRα/Akt/c-myc/cyclinD1 signaling was a major molecular pathway that drove the development of hepatomas after DEN induction. However, in WT livers, EGFR and MET signaling, rather than PDGFRα signaling, might be critical for DEN-induced hepatocarcinogenesis.

## Discussion

Various lines of evidence demonstrated that PDGFRα is a key factor in many pathophysiological processes.^17^ Previous study found that the expression level of PDGFRα was significantly elevated in the early stage of liver development and maintained at a lower level in adult normal livers.^2^, ^5^ When subjected to PH, the remnant liver would have a substantially increased expression of PDGFRα.^5^ Not surprisingly, overexpression of PDGFRα has been implied in the development and progression of various cancers, including HCC.^2^, ^6^, ^7^, ^9^ And the anti-PDGFRα treatment has been verified efficacy in these diseases.^2^, ^9^, ^18^, ^19^ As such, PDGFRα has been recognized as an “oncogene”.^1^ However, whether specific activation of PDGFRα in murine livers could necessarily promote liver regeneration and hepatocarcinogenesis remained unknown.

To address these questions, we are the first to generate hepatocyte-specific TG mice overexpressing PDGFRα*^D842V^* mutant. As known, D842V mutation (Exon 18 mutation) is the most frequent one among all the mutation types of PDGFRA in GIST, which renders PDGFRα protein stable and resistant to imatinib treatment.^20^-^22^ Unexpectedly, overexpression of PDGFRα*^D842V^* mutant in murine hepatocytes prevented liver regeneration after 2/3 PH, and inhibited DEN-induced hepatocarcinogenesis. As we further identified, downregulation of MET and EGFR signaling acted as an antagonistic mechanism with continuous activation of PDGFRα signaling, which subsequently retarded liver regeneration capacity after PH and chemically induced tumorigenesis in TG mice.

Intriguingly, there was no overt phenotype or any detectable abnormalities in TG mice overexpressing D842V mutant PDGFRα. In fact, the LW/BW ratio at 1 or 2-month was not significantly different among TG and WT mice. However, a continuous activation and overexpression of PDGFRα*^D842V^* led to the upregulation of total and phosphor-PDGFRα Y849, as well as its downstream target genes, such as Akt, cyclin D1 and c-myc, in 2-month-old TG versus WT livers. Increased PDGFRα was located mainly in the cell membrane and cytoplasm of the hepatocytes. These results confirmed the successful generation of a hepatocytes-specific PDGFRα*^D842V^* mutant overexpression mice model. It was noteworthy that another phosphorylated form of PDGFRα (total and phosphor-PDGFRα Y742) had been investigated with no changes. This may be because that the PDGFRα D842V mutant can lead to automatic activation of PDGFRα signaling, in which a ligand-free receptor Tyr-phosphorylation (Y849) can be activated instead of global expression of PDGFRα itself.

Despite overexpression of continuous activated PDGFRα*^D842V^* mutants in hepatocytes, the TG livers showed compromised regeneration capacity than WT livers. Interestingly, TG mice had a dramatic increase and activation of PDGFRα at 24 hours and subsequent decrease from 48 hours afterward, while WT mice had a gradual increase of PDGFRα, which was prominent at 72 hours after PH and even higher than TG livers (Figure 3C & D). In fact, overexpression of PDGFRα*^D842V^* mutants led to decreased expression of other tyrosine kinase receptors, such as MET and EGFR, which might account for a delayed liver regeneration among TG versus WT mice. In another study by Awuah and his colleagues, a hepatocytes-specific PDGFRα knockout mouse (KO) has been constructed.^5^ They found the absence of PDGFRα in hepatocytes resulted in compromised extracellular signal-regulated kinases and Akt activation at the early stage after PH, and subsequently delayed liver regeneration. However, compensatory increases of EGFR and MET expression then maintained later activation of Akt, which allowed for normal hepatocyte proliferation of KO mice at later stages.^5^ EGFR and Met expression are paramount during the process of liver regeneration and hepatocyte proliferation.^23^, ^24^ Combined systemic disruption of EGFR and Met signaling induced liver failure in normal mice.^25^ In fact, several previous studies have found that MET and EGFR could be activated within 30 to 60 minutes after PH. 26, 27 Hepatocytes are the first hepatic cells entering into DNA synthesis following PH under the activation of a large number of signaling pathways.^28^ Once activated, hepatocytes could secrete a variety of cytokines associated with cell mitoses, such as VEGF, TGFα, FGF, and PDGF, in an autocrine or paracrine manner.^26^, ^27^ During liver regeneration, these cytokines secreted by hyperplastic hepatocytes played an indispensable role. Liver regeneration was a redundant process, currently no evidences have been found that the elimination of any single gene after partial hepatectomy could reduce the ability of liver regeneration excepting inhibition of HGF/c-met signaling.^29^ The data of the present study suggests that compensatory downregulation of EGFR and MET after continuous activation of PDGFRα might be one of the pivotal signaling attributing to compromised liver regeneration after PH.

Hepatic fibrosis and cirrhosis as a result of chronic liver disease are precursors of HCC. Approximately 80% of HCC develop with underlying liver cirrhosis.^30^ We and others have demonstrated that PDGFRα was overexpressed and activated in a high proportion (~80%) of HCCs, 2, 5, 6, 8 In our previous liver-specific β-catenin knockout mice model, activation of PDGFRα signaling exacerbated hepatic inflammation, fibrosis, cirrhosis, and hepatocarcinogenesis.^7^ Perhaps not surprisingly, overexpression of PDGF-A and PDGF-C in the murine liver, both of which are well-known ligands of PDGFRα, induced liver fibrosis and tumorigenesis.^31^, ^32^ In our current study, overexpression of PDGFRα*^D842V^* mutants in TG mice induced higher expression of its ligands, such as PDGF-A and C, and subsequent more severe liver fibrosis than WT after DEN treatment. This finding further confirmed the activation of PDGFRα signaling possibly through paracrine mechanism in TG livers. Unexpectedly, continuous activation of PDGFRα in TG livers inhibited hepatocyte proliferation and apoptosis, as well as DEN-induced hepatocarcinogenesis. In PDGFRα TG tumors, downregulation of EGFR and Met have been verified compared to WT tumors, although activation of PDGFRα and its downstream targets, such as phosphor-Akt, c-myc and CyclinD1 in TG tumors was confirmed in these TG tumors. By utilization of this unique transgenic mice model, we demonstrated that overexpression of PDGFRα*^D842V^* and activation of PDGFRα signaling did not promote, but inhibit hepatocarcinogenesis possibly through compensatory downregulation of EGFR and MET signaling. As such, it is not surprising that the Phase II study of imatinib failed to demonstrate the effectiveness on treatment of unresectable or advanced HCC.^22^, ^33^

In the process of tumorigenesis, certain angiogenic factors like PDGFs, EGF, VEGFs and HGF are secreted by tumor cells in the microenvironment and can maintain the status of malignance via paracrine or autocrine methods.^34^, ^35^ EGFR and HGF/MET were commonly overexpressed in cirrhotic liver and HCC tissues, and associated with liver inflammation, fibrosis and subsequent hepatocarcinogenesis, as well as malignant invasion, metastasis, and poor prognosis.^36^-^40^ Although the association between PDGFRα and EGFR and MET has not been fully clarified, the current study provided strong evidence that overexpression of PDGFRα*^D842V^* mutant led to continuous activation of PDGFRα signaling, but, in turn, inhibition of EGFR and MET signaling, which prevented DEN-induced hepatocarcinogenesis in PDGFRα*^D842V^* TG mice. In fact, shRNA-mediated silencing of EGFR during liver regeneration was found to induce an increase of PDGFRα.^24^ Knockout of PDGFRα also caused activation of EGFR and MET signaling during liver regeneration and in mouse embryonic fibroblasts.^5^, ^41^ Taken together, these findings highlighted the mutual interaction and well balance of the existed signaling during liver pathophysiology.

In conclusion, continuous activation of PDGFRα*^D842V^* does not promote, but inhibits hepatic regeneration and hepatocarcinogenesis, possibly through compensatory downregulation of MET and EGFR. For the performance of global PDGFRα overexpressing in liver regeneration and chemically induced hepatocarcinogenesis mice model, further study should be investigated in the future.
