# Overexpression of Platelet-Derived Growth Factor Receptor Α *D842V* Mutants Prevents Liver Regeneration and Chemically Induced Hepatocarcinogenesis via Inhibition of MET and EGFR: Erratum

**DOI:** 10.7150/jca.140353

**Published:** 2026-08-17

**Authors:** Zhao-Qing Du, Jian Dong, Mu-Xing Li, Jian-Fei Zhang, Jian-Bin Bi, Yi-Fan Ren, Li-Na Zhang, Rong-Qian Wu, Satdarshan P.S. Monga, Yi Lv, Xu-Feng Zhang, Hai-Chen Wang

**Affiliations:** 1 Department of Hepatobiliary Surgery and Institute of Advanced Surgical Technology and Engineering, The First Affiliated Hospital of Xi'an Jiaotong University. Xi'an, Shaanxi Province, 710061, China;; 2 National-Local Joint Engineering Research Center for Precision Surgery & Regenerative Medicine, The First Affiliated Hospital of Xi'an Jiaotong University. Xi'an, Shaanxi Province, 710061, China;; 3 Department of General Surgery, Peking University Third Hospital, Beijing, 100083, China;; 4 Department of Surgical Oncology, Shaanxi Provincial People's Hospital, Xi'an, 710068, China;; 5 Department of Pharmacy, the Second Affiliated Hospital of Xi'an Jiaotong University, Xi'an, China;; 6 Department of Pathology and Medicine and Pittsburgh Liver Research Center, University of Pittsburgh, School of Medicine and University of Pittsburgh Medical Center, Pittsburgh, PA, USA;; 7 Department of Cardiovascular Surgery, The First Affiliated Hospital of Xi'an Jiaotong University. Xi'an, Shaanxi Province, 710061, China.

Following publication of the original article, the authors identified errors in Figure 4A. We carefully checked the original data and would like to amend it as following:

In Figure 4A, the 10-month-old wildtype mice liver was misused (right middle photo). The correct image is now provided. The original article remains unchanged except as noted above. This does not affect the primary outcomes of the article.

## Figures and Tables

**Figure 4A:**
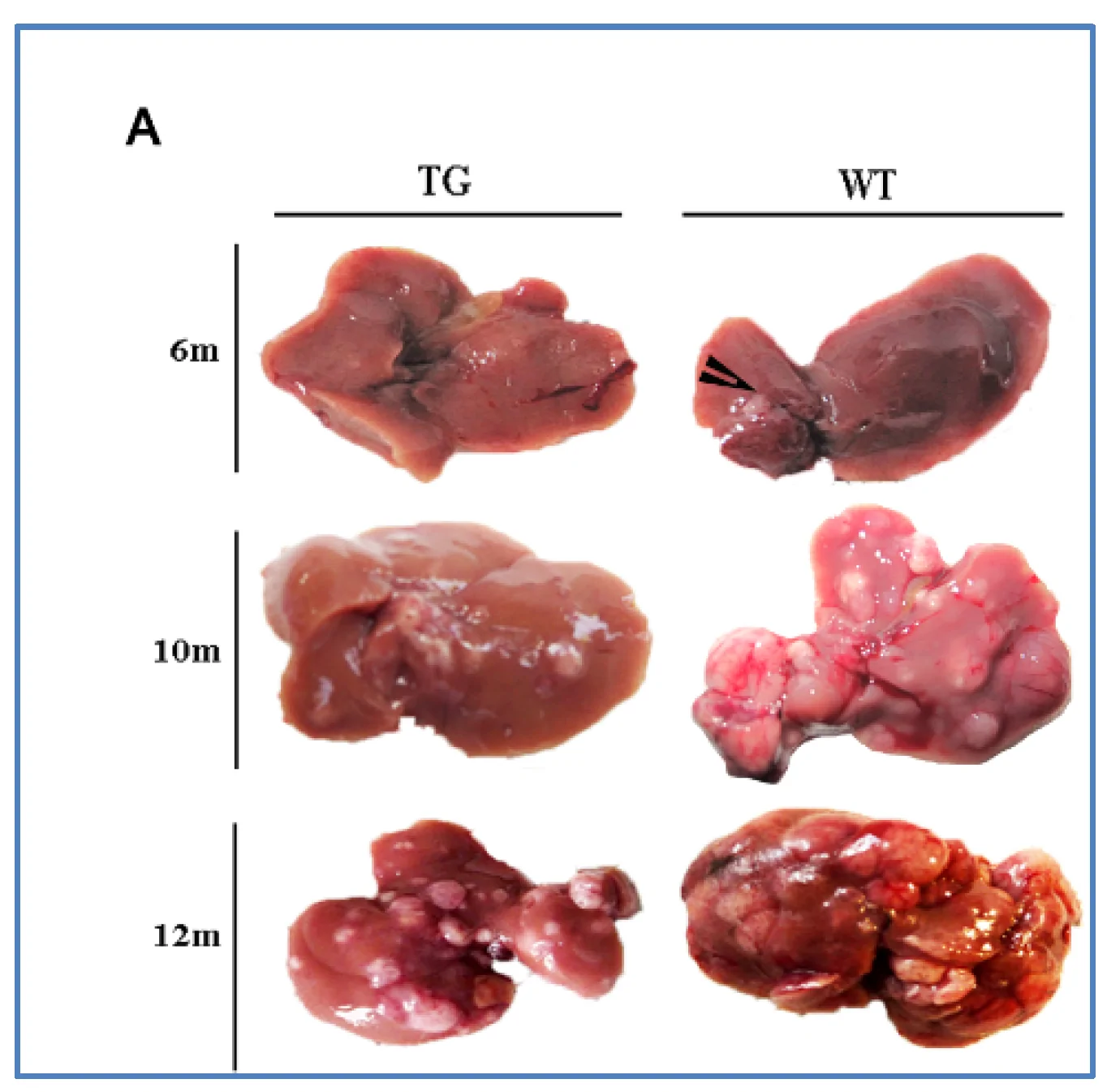
Correct image.

